# Accelerating Neoantigen Discovery: A High-Throughput Approach to Immunogenic Target Identification

**DOI:** 10.3390/vaccines13080865

**Published:** 2025-08-15

**Authors:** Lena Pfitzer, Gitta Boons, Lien Lybaert, Wim van Criekinge, Cedric Bogaert, Bruno Fant

**Affiliations:** 1 myNEO Therapeutics, 9000 Ghent, Belgiumlien.lybaert@myneotx.com (L.L.); cedric.bogaert@myneotx.com (C.B.); bruno.fant@myneotx.com (B.F.); 2 Department of Bioinformatics, Ghent University, 9000 Ghent, Belgium

**Keywords:** neoantigen, immunogenicity, machine learning, immuno-oncology, T cell

## Abstract

**Background**: Antigen-targeting immunotherapies hinge on the accurate identification of immunogenic epitopes that elicit robust T-cell responses. However, current computational approaches focus primarily on MHC binding affinity, leading to high false-positive rates and limiting the clinical utility of antigen selection methods. **Methods**: We developed the neoIM (for “neoantigen immunogenicity”) model, a first-in-class, high-precision immunogenicity prediction tool that overcomes these limitations by focusing exclusively on overall CD8 T-cell response rather than MHC binding. neoIM, a random forest classifier, was trained solely on MHC-presented non-self peptides (n = 61.829). Its performance was assessed against that of currently existing alternatives on several in vitro immunogenicity datasets. In addition, its clinical impact was investigated in two retrospective analyses of clinical trial data by assessing the effect of neoIM-based antigen selection on the positive immunogenicity rate of personal vaccine designs. Finally, the potential for neoIM as a biomarker was investigated by assessing the correlation between neoIM scores and overall survival in a melanoma patient cohort treated with checkpoint inhibitors (CPI). **Results**: neoIM was found to substantially outperform publicly available tools in regards to in vitro benchmarks based on ELISpot assays, with an increase in predictive power of at least 30%, reducing false positives and improving target selection efficiency. In addition, using neoIM scores during patient-specific antigen prioritization and selection was shown to yield up to 50% more clinically actionable antigens for individual patients in two recent clinical trials. Finally, we showed that neoIM could further refine response prediction to checkpoint inhibition therapy, further demonstrating the importance of evaluating neoantigen immunogenicity. **Conclusions**: These findings establish neoIM as the first computational tool capable of accurately predicting epitope immunogenicity beyond MHC affinity. By enabling more precise target discovery and prioritization, neoIM has the potential to accelerate the development of next-generation antigen-based immunotherapies.

## 1. Introduction

Cancer immunotherapy aims to re-activate or boost a patient’s own immune system to specifically recognize and eliminate tumor cells [1]. While early cancer vaccine trials targeting tumor-associated antigens (TAAs) saw limited efficacy, this shortfall can be traced to immune tolerance mechanisms against self-antigens, as well as potential systemic toxicity issues [2,3,4,5]. In contrast, neoantigens, which arise from tumor-specific mutations, bypass central tolerance and are therefore more likely to trigger robust, safe antitumor responses [6,7,8]. Indeed, clinical successes with vaccines targeting patient-specific neoantigens [9,10,11] and the emergence of resistance via neoantigen loss [12] underscore the centrality of neoantigens in modern immunotherapies.

Despite improved genomic profiling tools that reveal a vast landscape of candidate neoantigens, only a small subset elicits a clinically meaningful T-cell response. Recent evidence from the Tumor Neoantigen Selection Alliance (TESLA) shows that just 6% of top-predicted peptides are confirmed as immunogenic [13], a finding echoed by multiple groups [14,15,16]. This highlights the urgent need for more accurate, high-throughput methods to pinpoint truly immunogenic neoantigens.

While in vitro assays like ELISpot [17] can validate neoantigen immunogenicity, time and resource constraints make them prohibitive for large-scale screening in personalized settings. Most computational pipelines currently prioritize based on MHC-I binding affinity, yet numerous studies show that binding alone does not guarantee a T-cell response [14,18]. Additional physicochemical features, binding stability, expression levels, and other factors are increasingly recognized as important [19,20,21,22,23,24,25]. However, existing immunogenicity predictors remain limited, often conflating peptide presentation likelihood with true T-cell recognition.

To address this gap, we developed neoIM, an MHC-allele-agnostic tool trained exclusively on presented peptides to decouple presentation from immunogenicity. By focusing on non-self peptides validated as immunogenic or non-immunogenic, neoIM aims to refine tumor-specific TCR recognition signals beyond the sole determination of binding affinity. Here, we describe the model’s design, systematically validate its performance against that of other tools, and demonstrate its impact in differentiating high-confidence neoantigens from diverse sources of genomic variation and in clinical contexts. We also highlight how neoIM-predicted immunogenic burden correlates with response to checkpoint inhibitors, suggesting an additional biomarker utility for tumors with low mutational burden.

## 2. Methods

### 2.1. Assembly of the Training Datasets

Positive dataset: To compile a set of immunogenic peptides, peptides were collected from the Immune Epitope Database (IEDB) (version 5, January 2025) that were defined as “linear peptide”, tested in Homo sapiens, annotated as MHC Class I, and had a “positive” qualitative measurement indicating a T-cell response. Only peptides of 9–11 amino acids in length were retained. Any peptide matching the human reference proteome (UniProt ID: UP000005640) or present in duplicate was removed, yielding 10,069 unique immunogenic peptides.

Negative dataset: For non-immunogenic but MHC-presented peptides, we again used IEDB (version 5, January 2025), selecting entries under the same “linear peptide”, Homo sapiens, MHC Class I, and 9–11 amino acid criteria. However, these peptides were only recorded as “positive” for MHC presentation (i.e., binding), without evidence of a positive T-cell assay. To ensure non-self status, we removed any peptide fully matching the reference proteome or overlapping with known immunogenic peptides and retained those carrying 1–3 mismatches to the human proteome. This process produced 51,760 putative non-immunogenic peptides, allowing a 1:3.5 ratio of positive to negative instances while preserving length distributions.

### 2.2. Characterization of the Training Datasets

We compared the amino acid distributions between immunogenic (Set A) and non-immunogenic (Set B) MHC-presented peptides focusing on 9-mers, as they represent the largest subset (69%) in the dataset. Chi-square tests were performed at each position to identify significant differences in residue composition, followed by Benjamini–Hochberg correction for multiple testing. We computed absolute frequency differences between Set A and Set B and used Shannon entropy to quantify per-position sequence variability. To visualize amino acid distribution shifts, absolute frequency difference logos were generated using the logomaker Python package (v0.8) [26], where positive values indicate enrichment in Set A, and negative values indicate enrichment in Set B. In addition, we computed the per-position Shannon entropy for each peptide set using the natural logarithm as a measure of amino acid diversity. Entropy values thus ranged from 0 (complete conservation) to ln(20) (~3, maximal diversity), allowing us to quantify the variability of each position across the two sets.

To ensure that negative peptides were primarily truly non-self, we checked for overlaps with known single-nucleotide polymorphisms (SNPs) using TransVar and dbSNP build 151.

### 2.3. Peptide Encoding

We retrieved 553 amino acid physicochemical properties from AAindex1, removed 13 properties with missing values, and z-scaled the remaining 540 features. Principal component analysis (PCA) was then applied; the first 10 PCs (explaining ~89% of variance) were retained. Each amino acid was encoded into a 10-dimensional vector, yielding an ordered 90–110-dimensional representation for each 9–11mer (with zero-imputation for shorter peptides). Additional peptide-level features were computed via Bio.SeqUtils.ProtParam (v1.75) [27], including molecular weight; aromaticity; instability index; isoelectric point; GRAVY (grand average of hydropathy); and the fraction of amino acids predicted to be in helix, turn, or sheet conformations (secondary_structure_fraction), resulting in a final 118-dimensional feature vector per peptide.

### 2.4. Algorithm Training

After assembling the training datasets (positive immunogenic peptides and negative non-immunogenic, yet MHC-presented peptides), we trained a random forest classifier [28] with 10-fold cross-validation. Early developments benchmarks showed that the random forest classifier outperformed other classification methods (regression, gradient boosting, SVMs, and deep learning or neural network-based architectures). This can likely be explained by the limited number of features used during modeling, limiting the added effectiveness that more recent elaborate deep learning architectures could bring, and by the relatively limited number of data points available per feature used, which limits the usability of neural networks in general. The final model used 1000 trees, a minimum samples-split of 3, Gini-based splits, and no bootstrap sampling. The model outputs an immunogenicity score from 0 to 1, with higher values indicating stronger immunogenic potential. Feature importance was extracted from the fitted model.

### 2.5. Bias Testing for HLA and Peptide Length

To evaluate whether neoIM’s immunogenicity predictions are influenced by HLA allele identity or peptide length, we performed bias testing using a dataset of 100,000 peptides randomly sampled from the human proteome in equal proportions of 9-, 10-, and 11-mers. MHC presentation was predicted using MHCnuggets (v2.3) [29] for 38 HLA-A, 52 HLA-B, and 12 HLA-C alleles. For each HLA allele, the top 2% of peptides with the highest predicted binding affinity (rank (%) < 2) were selected as presented peptides. neoIM immunogenicity scores were then calculated for each presented peptide. To assess potential biases, peptides were categorized by their HLA allele and length, and Kruskal–Wallis tests were applied to detect significant differences in immunogenicity score distributions. Effect sizes (η^2^) were computed to quantify the contribution of these factors to score variance. For visualization, immunogenicity score distributions were plotted at the HLA supertype level (HLA-A, HLA-B, HLA-C), with HLA-A*02:01 highlighted separately to assess potential overrepresentation effects.

### 2.6. Benchmarking

We compared neoIM to three other tools—IEDB immunogenicity predictor (https://nextgen-tools.iedb.org/pipeline?tool=tc1, accessed in 1 January 2025) [30], the antigen.garnish dissimilarity score (v2.3.1) [31], and PRIME (v2.0) [32]—using an independent dataset of 540 peptides (9–11-mers) obtained from the literature [33,34] (Table 1). INeo-Epp [35] was not included in this benchmarking because its training data includes a significant portion of the benchmarking dataset, and the results would therefore be heavily biased. All peptides were verified as MHC-presented by NetMHCpan 4.0 (rank < 2); 67 peptides were experimentally confirmed immunogenic, and 534 were not. Of the 534 peptides, however, 92 were recorded to result in a positive immunogenicity assay in other publications in the IEDB database and were therefore excluded from the analysis, resulting in 442 peptides. We plotted ROC and PR curves for each method, treating the 67 immunogenic peptides as true positives and the 442 non-immunogenic peptides as true negatives.

### 2.7. In Vitro Immunogenicity Validation with ELISpot

To test immunogenicity across a diverse range of mutation types and neoIM scores, we selected 33 candidate peptides (SNVs, indels, gene fusions, retained introns, and long non-coding RNAs) observed in real tumor data. Each peptide was required to be classified as being presented by at least two HLA alleles (via MHCnuggets) of each of the three available donors. Cultured monocytes were differentiated into immature dendritic cells (iDCs) using a cytokine cocktail, and were then further matured. The DCs were loaded with test peptide pools (five different pools). After loading, the DCs were co-cultured with autologous pan T cells for 8 days in the presence of a cytokine cocktail. During co-culture, the medium and cytokines were regularly refreshed. After 8 days, the pan T cells were harvested and re-stimulated with peptide pool-loaded monocytes (round 1) and co-cultured for another 8 days in the presence of a cytokine cocktail (round 2). After round 1 and round 2, the pan T cells were harvested and re-stimulated with peptide pool-loaded monocytes into IFN-γ FluoroSpot plates. After overnight stimulation, The IFN-γ FluoroSpot plates were developed according to the manufacturer’s protocol. The number of IFN-γ secreting pan T cells was measured using the Mabtech IRIS™ FluoroSpot Reader and analyzed via the non-parametric distribution free resampling (DFR) method (see Table S1 for raw results). Wells were scored positive if they produced ≥25 spot-forming units above background and at least a twofold increase relative to the results for the DMSO-only controls. Any peptide meeting these criteria in at least one donor was deemed immunogenic, and these outcomes were then compared to neoIM scores (calculated prior to peptide selection). For the ROC and lift curves, predictions for each peptide via the neoIM algorithm were compared to several immunogenicity predictors: the IEDB immunogenicity predictor [30], the antigen.garnish [31] dissimilarity score, PRIME [32], and INeo-Epp (http://www.biostatistics.online/dbPepNeo/toolINE.html, accessed in 1 January 2025) [35]. As both PRIME and INeo-Epp scores are dependent on a specific HLA allele, the PRIME strategy for multiallelic data was used for both predictors, which takes the score predicted for the HLA allele with the lowest binding affinity into account.

### 2.8. Retrospective Analysis of Personalized Cancer Vaccine Trials

Patient-specific molecular information and per-epitope immunogenic response to the vaccine were extracted from two recent vaccine trial populations [36,37]. In a first step, for each patient (n = 19 in pancreatic cancer [36], n = 12 in melanoma [37]), the exact set of epitopes (n = 20, optimally, in pancreatic cancer, n = 10 in melanoma) selected for vaccine design during the trial was gathered, and the true positive hit rate of each vaccine was computed as the number of epitopes for which an immune response could be shown (as evaluated by the original papers, using ex vivo ELISpot assays in both cases) over the total number of epitopes selected. In a second step, starting for each patient from the same set of mutations, neoIM was used to prioritize epitopes; for each patient, the top n epitopes were selected according to the neoIM score (n being the number of epitopes present in the patient’s original formulation, for side-to-side comparison purposes), and the true positive hit rate using this method was computed as well. Paired z-score testing was performed to determine which method was the more efficient—for each patient, a pair was considered to be, on the one hand, the percentage of immunoreactive epitopes in the final vaccine design, as chosen in the original paper, and on the other hand, the percentage of immunoreactive epitopes in the final vaccine design, as chosen using neoIM scores.

### 2.9. Biomarker Analysis of a CPI-Treated Cohort

The potential of neoIM-derived immunogenicity prediction as a prognosis biomarker for response to immunotherapy was tested in a cohort of 64 melanoma patients treated with CPI therapy, and the results were compared to other biomarkers [38]. For each patient in this cohort, several metrics were determined to study their correlation to therapy outcome. First, the number of mutations per patient was extracted from the genomic variation data, forming the TMB-related biomarker. Next, the number of presented peptides resulting from non-synonymous mutations, i.e., rank (%) < 2, was computed using MHCnuggets, forming the (MHC) presentation-related biomarker [29]. Finally, the neoIM algorithm was used to assess the immunogenicity of a patient’s tumor by computing the neoIM-max–mean value as a general measure of immunogenicity. The value is computed by calculating the average neoIM score across all possible 9–11-mers covering a specific mutation, and selecting the highest average score across all the mutations in the tumor. The mean–max value thus represents the immunogenicity of the most immunogenic region in a tumor, and is further referred to as the immunogenicity-related tumor biomarker.

The cohort was first separated into two groups based on a previously reported, biologically relevant threshold of 100 mutations per exome. For either group thus obtained, patients were further divided at the median value of the TMB-, presentation- or immunogenicity-related biomarker to obtain two evenly sized groups, for which overall survival (OS) was compared and visualized in Kaplan–Meier curves. Statistical testing was performed using the logrank test.

## 3. Results

### 3.1. Building the Training Dataset of the Immunogenicity Model

**Figure 1:**
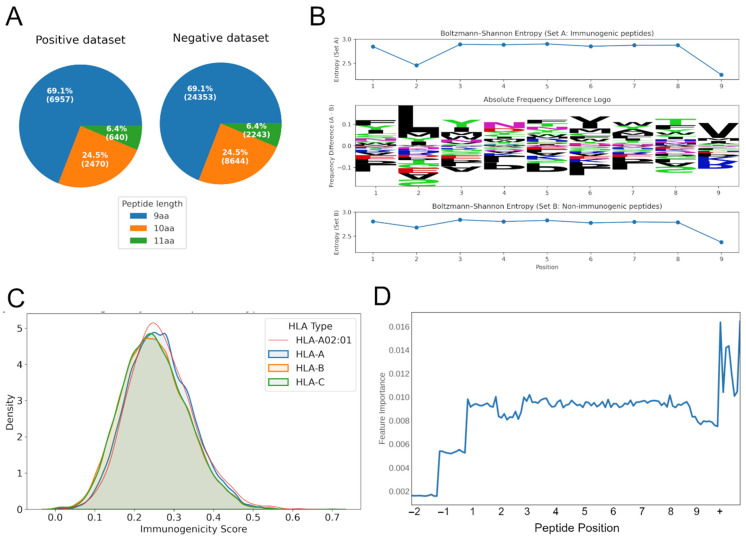
Characteristics of the training data and model training. (**A**) The distribution of peptide lengths for the positive and negative training dataset. (**B**) Absolute frequency difference logo (center) showing amino acid composition shifts between immunogenic (Set A) and non-immunogenic (Set B) peptides. Positive values indicate enrichment in Set A, while negative values indicate enrichment in Set B. Per-position Shannon entropy (top and bottom). (**C**) Density distribution of immunogenicity scores across HLA supertypes (HLA-A, HLA-B, HLA-C). The highlighted red line represents the distribution for HLA-A02:01. (**D**) The impact of features related to a specific position in the peptide (10 features per peptide position, positions −1 and −2 only relevant for peptides of length 10 or 11 amino acids) or peptide-wide (+) on model performance. aa, amino acids.

Since our objective was to predict which MHC-presented peptides elicit a T-cell response, we focused on assembling two datasets of experimentally validated MHC binders. The positive dataset comprised 10,069 peptides (9–11 amino acids) that exhibited at least one mismatch from the human reference proteome and were shown to elicit a T-cell response. The negative dataset contained 35,241 peptides (maintaining similar length distributions to those in the positive set, see Figure 1A) that were likewise confirmed MHC binders but showed no evidence of T-cell reactivity. These peptides carried 1–3 mismatches compared to the results for the human reference proteome, making them highly likely to be derived from mutations. A check against known single nucleotide polymorphisms (SNPs) confirmed that only a small fraction (<5%) might represent common variants. Hence, the negative set primarily consisted of non-self and non-immunogenic peptides.

By compiling both immunogenic and non-immunogenic peptides validated to bind MHC, we aimed to build a model focusing exclusively on the features that differentiate truly immunogenic peptides—i.e., those capable of initiating a T-cell response—from non-immunogenic, yet MHC-presented peptides. This design thus prevents the model from simply learning MHC binding affinity and instead directs it toward identifying TCR-relevant properties. A comparison of amino acid usage between these sets showed significant differences in amino acid composition between immunogenic (Set A) and non-immunogenic (Set B) peptides (*p*-adjusted < 0.05) at all positions. Among them, positions 1, 5, and 7 exhibited the largest absolute frequency differences, suggesting that these sites offer the greatest contribution to distinguishing immunogenic from non-immunogenic peptides (Figure 1B, center). Overall, both sets showed high entropy values (2.8–2.9) across most positions, consistent with broad amino acid diversity. Notably, positions 1 and 5 exhibited the largest differences, indicating stronger constraints on these sites in immunogenic peptides compared to non-immunogenic peptides (Figure 1B, top and bottom). Position 1 showed the highest overall shift in composition, with a total frequency difference of 0.38 between the two sets. Entropy analysis revealed that positions 1 and 5 exhibited the largest differences in sequence variability, with Set B showing greater diversity at these sites. This suggests that these positions may be more structurally constrained in immunogenic peptides compared to in their non-immunogenic counterparts.

### 3.2. neoIM Model Training and Performance

Using only peptides experimentally validated as MHC-presented, we trained an HLA-agnostic random forest model, neoIM, to predict immunogenicity based on physicochemical properties. Each peptide was encoded using a combination of residue-level and peptide-level features including hydrophobicity, charge, and secondary-structure tendencies. Feature-importance analysis confirmed that both individual positions (except imputed zeros for shorter peptides) and global descriptors contributed to classification (Figure 1D). Furthermore, positions 2 and 9 (relative to 9 amino acid-length peptides), which are known to be relevant for presentation on MHC alleles, show reduced feature importance. Finally, analysis of the relative importance of individual features showcased a strong effect of hydrophobicity, among other features, on immunogenicity, especially at and around the anchor positions (Supplementary Figure S1), an observation shared by other studies in the field [13].

The dataset is composed of immunogenic (positive) and non-immunogenic (negative) peptides with a 1:3.5 positive-to-negative ratio. This class imbalance is typical in immunogenicity studies, where only a small fraction of presented peptides elicits a T-cell response. To account for this, we evaluated model performance using both ROC AUC and precision–recall (PR) AUC, as PR curves provide a more informative measure when the positive class is relatively rare.

**Figure 2:**
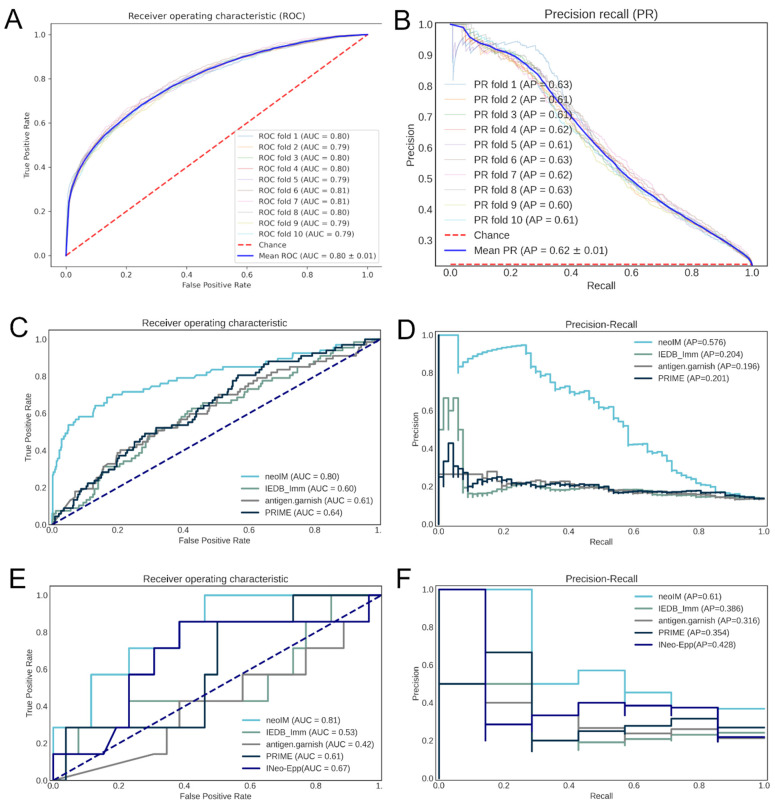
Correlation of various predictors with peptide immunogenicity. (**A**) ROC curve of the performance of the neoIM model on its cross-validation datasets; (**B**) precision-recall curve of the performance of the neoIM model on its cross-validation datasets; (**C**) ROC curve of the performance of neoIM and three other immunogenicity predictors on an independent validation dataset (n = 509 peptides); (**D**) precision-recall curve of the performance of neoIM and three other immunogenicity predictors on an independent validation dataset; (**E**) ROC curve of the performance of neoIM and three other immunogenicity predictors on n = 33 cancer-derived peptides; (**F**) performance-recall curve of the performance of neoIM and three other immunogenicity predictors on n = 33 cancer-derived peptides. In (**C**–**F**), light blue representes neoIM, green represents the IEDB immunogenicity predictor, grey represents antigen garnish, and dark blue represents PRIME.

In 10-fold cross-validation, neoIM achieved an ROC AUC of 0.80 and a PR AUC of 0.62 (Figure 2A,B), demonstrating high predictive performance. Because all peptides in the dataset were experimentally confirmed to be MHC-presented, neoIM specifically predicts the likelihood of T-cell recognition rather than MHC binding. As a result, it should be applied after a robust MHC presentation predictor to prioritize immunogenic candidates.

### 3.3. Bias Testing Demonstrates Minor Influence of HLA Allele and Peptide Length

Analysis of 2000 randomly sampled peptides predicted to be MHC-presented on several HLA alleles (38 HLA-A, 52 HLA-B, 12 HLA-C alleles) revealed that neoIM scores varied slightly across HLA alleles (H = 26,319.33, *p* < 0.0001), but the overall effect size was small (η^2^ = 0.1354), indicating that HLA allele identity explains only ~13.5% of the score variance. Similarly, peptide length showed a statistically significant effect (H = 2288.65, *p* < 0.0001), but the effect size was even smaller (η^2^ = 0.0095), confirming that peptide length has a negligible influence on predicted immunogenicity. Figure 1C illustrates score distributions at the HLA supertype level (HLA-A, HLA-B, HLA-C), with HLA-A*02:01 following the general trend of other HLA-A alleles, suggesting no substantial bias toward this commonly studied allele. While these variations are statistically significant, it remains unclear whether they reflect true biological differences in immunogenicity across HLA alleles and peptide lengths or subtle biases in the training data. Nevertheless, the small effect sizes indicate that neoIM’s predictions remain robust across diverse HLA contexts, reinforcing its broad applicability for neoantigen screening.

### 3.4. Benchmarking the neoIM Model

We next evaluated neoIM on two external datasets, comprising (i) viral peptides and (ii) tumor-derived neoantigens, all confirmed to be MHC-presented. The final set included 509 peptides, 67 of which were immunogenic (true positives), and 442 were non-immunogenic (no evidence for immunogenicity). Compared to other available tools (IEDB immunogenicity, antigen.garnish, PRIME, and INeo-Epp), see Table 2, neoIM delivered superior accuracy, with an ROC AUC of 0.80 versus 0.60–0.64 for the next-best methods (Figure 2C). Precision–recall analyses mirrored this trend, with neoIM reaching an AP of 0.58 compared to ~0.20 for the other predictors (Figure 2D).

### 3.5. ELISpot Validation of neoIM Immunogenicity Predictions for Different Neoantigen Types

To further test neoIM in the context of diverse cancer mutations, we performed ELISpot assays on 33 neoantigen events derived from cancer-specific genomic events observed in real-world tumor data. All 33 peptides were confirmed to be MHC-presented and were chosen to span a range of neoIM scores. Seven peptides (21%) elicited a positive T-cell response in at least one healthy donor, matching rates commonly reported in the literature [13,14,39]. The ROC curve for neoIM in this dataset yielded an AUC of 0.81, surpassing the 0.48–0.67 range for other predictors (Figure 2E). Moreover, 71% of the positive peptides were among the top 30% of scores, suggesting that neoIM can substantially reduce experimental burden by discarding lower-scoring peptides. Taken together, these results confirm that neoIM accurately predicts which presented peptides are truly immunogenic, significantly outperforming existing methods and offering a practical tool for prioritizing neoantigens for vaccine design or other immunotherapies.

Panels A and B show the neoIM receiver operator characteristic (ROC) (A) and precision–recall (PR) (B) curves resulting from 10-fold cross-validation. Panels C and D show the ROC (C) and PR (D) curves resulting from benchmarking on an external dataset of viral peptides. Panels E and F show the ROC (C) and PR (D) curves resulting from benchmarking based on the ELISpot results of 33 neoantigen peptides which have all been predicted to be present by netMHCpan4.0 and MHCnuggets.

### 3.6. Retrospective Analysis of Recent Personalized Cancer Vaccine Trials

**Figure 3:**
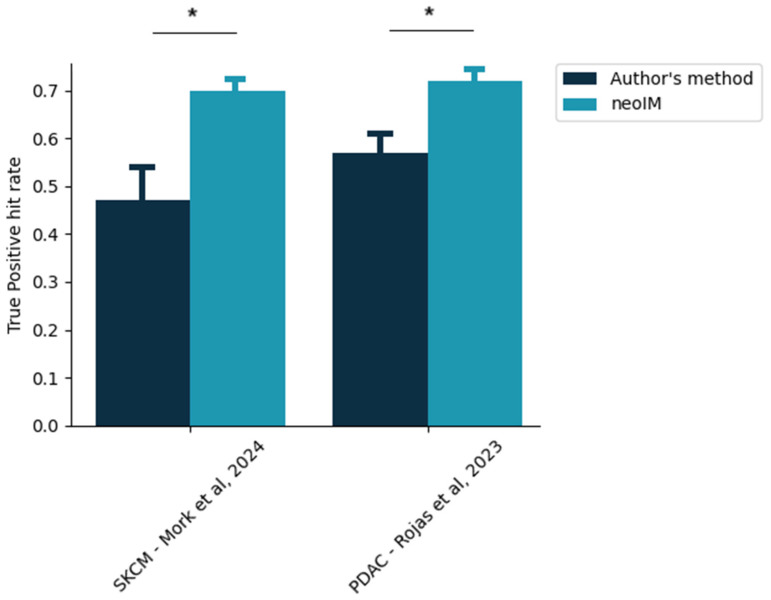
Proportion of clinically actionable epitopes in a given personalized vaccine design. The exact, patient-specific vaccine designs were extracted from two recent studies [28,36], and the proportion of selected epitopes yielding a positive CD8 immune response, according to the author’s method of selection, was thus computed (dark blue bars). In comparison, the same proportion as would be obtained exclusively by neoIM-driven selection is shown. Stars indicate *p* < 0.05 (z-score testing).

Personalized cancer vaccines have recently been shown to display the ability to provide measured clinical benefits for various indications [7,31]. However, even in such a clinically favorable setting, 53% (resp., 44%) of the epitopes administered to each melanoma (resp., pancreatic cancer) patient failed to raise any type of CD8^+^ immune response (Figure 3). In contrast, a neoIM-guided patient-specific epitope selection yields much more efficacious formulations. When the top epitopes as scored by neoIM were selected per patient, the proportion of actually immunogenic epitopes in any vaccine design was 70%, on average, in melanoma (versus. 47% in the original study) and 71% in pancreatic cancer (versus. 56% in the original study). This demonstrates neoIM’s superiority in the context of epitope selection for personalized vaccine design.

### 3.7. neoIM Tumor Immunogenicity as a Predictive Biomarker for CPI Treatment

In addition to PD-L1 expression, one of the most commonly used clinical biomarkers for response to CPI therapy is tumor mutational burden (TMB). Although these markers provide significant value in patient stratification, there is a sizable portion of patients for which the correlation between PD-L1 expression or TMB and CPI therapy response is not valid. For these patients, TMB or PD-L1 expression are insufficient as biomarkers, and other biological parameters should be investigated to better distinguish potential responders from non-responders.

Here, we evaluated whether the immunogenic potential of neoantigens, as estimated by neoIM, could enhance or complement traditional biomarkers for CPI response. For this purpose, we analyzed a cohort of 64 malignant melanoma patients treated with anti-CTLA-4 therapy and for which tumor-normal whole-exome sequencing data and clinical data were available. Snyder et al. [38] showed that a high number of neoantigen-producing mutations, defined as >100 mutations—a threshold that has been commonly reported for melanoma studies—indeed correlated with improved clinical benefit of CTLA-4 blockade in this cohort. In contrast, it failed to correctly identify a subset of patients with a low mutation rate (<100 ns mutations) who showed a positive response to CPI therapy. This suggests that solely relying on the number of ns mutations might not capture all characteristics for the prediction of a positive response to immunotherapy in patients with low TMB tumors. This highlights the need for additional metrics to ensure that no potential responder is overlooked.

**Figure 4:**
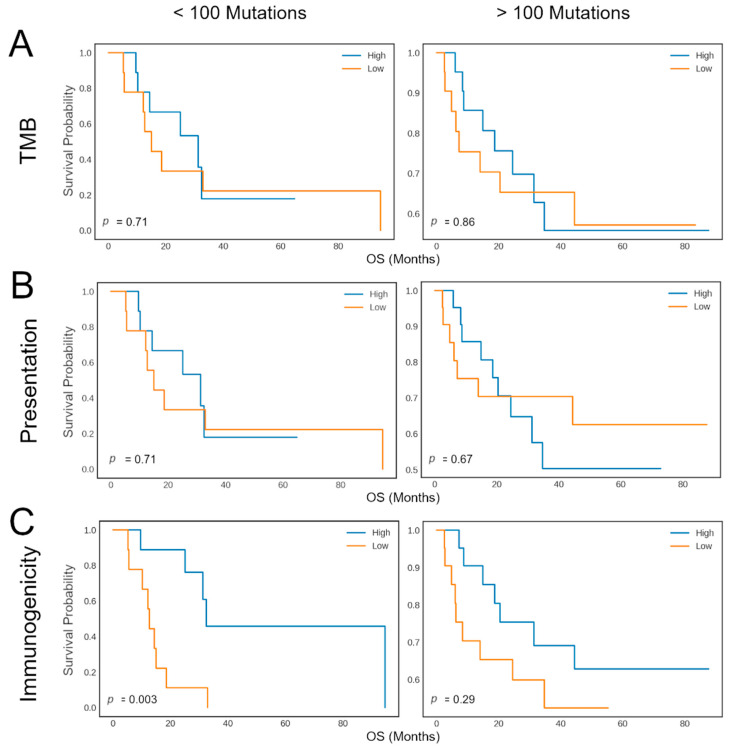
Kaplan–Meier plots showing the response rate (y-axis) versus the response duration (x-axis) of melanoma patients during CTLA-4 blockade. The survival curves are shown for patients with less than 100 mutations (**left**) and patients with more than 100 mutations (**right**). Patients are then further divided into two groups, indicated as “High” (blue) or “Low” (orange), according to the median number of mutations (**A**), the number of presented mutations (**B**), or based on the immunogenicity-related tumor biomarker as computed by neoIM (**C**).

For this purpose, we investigated whether tumor immunogenicity, as evaluated by neoIM, could help refine patient classification. We stratified patients into groups of high mutation rate (>100 mutations per exome) versus low mutation rate (≤100 mutations per exome). Then we assessed overall survival (OS) in the subgroups using a median split on overall TMB, number of presented peptides (MHCnuggets), and the immunogenicity-related tumor biomarker as computed by neoIM (MHCnuggets + neoIM) (Figure 4). As expected, for patients with more than 100 ns mutations (n = 48), the duration of response was similar across groups. However, among the subset of patients with fewer than 100 ns mutations (n = 16), a significant difference in OS was observed based on tumor immunogenicity as predicted by neoIM. Interestingly, patients with high tumor immunogenicity experienced notably longer survival (*p*-value = 0.003). Further stratification based on TMB or number of presented peptides did not result in a significant difference in OS (*p*-values = 0.78 and 0.86, respectively). This underscores tumor immunogenicity as a pivotal factor in CPI response and highlights the potential of neoIM as a biomarker for CPI therapy response prediction, especially in low TMB tumors. Such tumors, despite displaying a low mutation count, can still harbor a significant load of highly immunogenic neoantigens, which can stimulate specific T-cell responses that can, in turn, be amplified with CPI therapy.

## 4. Discussion

Identifying the fraction of actionable, truly immunogenic tumor antigens remains a major challenge in cancer immunotherapy. Most current computational approaches rely primarily on MHC binding affinity as a predictor of immunogenicity, leading to an overwhelming number of false positives—a limitation that has direct clinical consequences. Indeed, data from clinical neoantigen vaccine trials indicate that, despite selecting multiple predicted neoantigens per patient, only 1–2 peptides per vaccine typically induce measurable T-cell responses (e.g., BioNTech’s iNeST trial NCT03289962 reported a median of 1 responding neoantigen per patient [36], while Ott et al. observed a median of 2 per patient [14]). This highlights the urgent need for more precise selection criteria to avoid wasting resources on non-immunogenic targets.

Here, we present neoIM, a first-in-class predictor designed to decouple MHC presentation from T-cell recognition by focusing on physicochemical features that drive true immunogenicity. By training exclusively on peptides experimentally confirmed to be MHC-presented, neoIM eliminates the major confounding factor of peptide presentation likelihood, allowing it to capture key determinants of TCR engagement. This sets it apart from other tools like PRIME [30], INeo-Epp [35], and DEEPimmuno [40], which remain influenced by MHC binding properties and thus inherit the limitations of affinity-based selection.

Bias testing confirmed that neoIM predictions remain robust across diverse HLA alleles and peptide lengths. Using a dataset of 75,000 MHC-presented peptides predicted by MHCnuggets across 98 alleles, we observed only modest score variations between different HLA alleles (η^2^ = 0.1354) and negligible effects of peptide length (η^2^ = 0.0095). The distribution of scores for HLA-A*02:01 followed the general trend of other HLA-A alleles, confirming no strong overrepresentation bias. These findings reinforce neoIM’s applicability across diverse MHC contexts, making it a more broadly useful tool for prioritizing immunogenic peptides. In both cross-validation and external benchmarking, neoIM outperformed existing immunogenicity predictors (AUC ~0.8), demonstrating superior precision in identifying true immunogens. Furthermore, in vitro ELISpot assays on 33 diverse cancer-derived peptides confirmed that neoIM accurately enriches for immunogenic neoantigens, dramatically improving the efficiency of candidate selection.

Some limitations of neoIM include an exclusive focus on 9–11-mers, even though it is known that MHCI molecules can bind to epitopes anywhere from 8 to 14 amino acids in length. However, 9–11-mers account for 85% of the MHCI ligandome and thus, of the set of potentially actionable peptides; focusing on predicting their immunogenicity allows for the development of a model that remains usable for a large majority of MHCI-presented epitopes while limiting the noise introduced by minority classes like 12–15-mers.

Another limitation is that neoIM predicts a general CD8 T-cell response without providing the exact TCRs responsible for the reaction. In contrast, other predictors, such as NetTCR, will predict interaction likelihoods between given TCR sequences and specific epitopes, reaching a higher level of resolution. However, such predictors are often hampered by the relative lack of TCR-antigen-specific training data, and often pay for their higher resolution with lower performance. While neoIM cannot immediately identify potentially immunoreactive TCRs, limiting its direct applicability in e.g., diagnostics, it achieves better predictive power in assessing the likelihood of T-cell immune reaction, a more directly clinically applicable effect.

Finally, another limitation is that the model currently ignores indication-specific features; future iterations of the model will focus on investigating differences in immunogenicity between different primary cancer tissues.

Looking forward, the model’s utility could be further expanded by incorporating additional experimental data, particularly for longer peptides relevant to MHC class II presentation and CD4+ T-cell responses, which play critical roles in anti-tumor immunity.

neoIM’s ability to reliably predict epitope immunogenicity offers direct clinical applications, as demonstrated by the retrospective analysis performed in this study. Using neoIM to select epitopes on a per-patient basis increases the actual clinically actionable fraction of any vaccine design by a factor of up to 50%. This efficiency is critical for several reasons; on the one hand, therapeutic strategies aiming at delivering a cancer vaccine payload often have an upper limit for payload size. It is therefore imperative to maximize the efficacy of every epitope contained in a vaccine formulation and to minimize wasted space. On the other hand, single-epitope responses are often insufficient to lead to consistent tumor regression. Various mechanisms of immune escape, such as HLA loss of heterozygosity or neoepitope expression silencing, allow tumors to easily circumvent tumor-lysing responses mounted against single epitopes. It is therefore essential for any vaccine to trigger a multi-pronged immune response, targeting several neoepitopes and relying on different HLA alleles; therefore, neoIM’s ability to maximize the number of clinically relevant epitopes in a given vaccine is critical to future therapy successes.

Currently, TMB, as a proxy for tumor immunogenicity, is used to select patients most likely to benefit from CPI treatment. Using high TMB as an inclusion criterion for CPI is a valid strategy, as these patients tend to respond well to therapy, but stratification is suboptimal in patients with low TMB [7,22,38]. In a cohort of 64 CPI-treated melanoma patients, Snyder et al. [38] proposed a neoantigen-based signature which resulted in a slightly improved classification by further excluding high-mutation-rate patients unlikely to respond. By using neoIM to rate the immunogenic potential of mutations, patients with low mutation rates can, in turn, be further divided into groups of low-mutation, high-immunogenicity patients and low-mutations, low-immunogenicity patients; the former group displays a significantly longer overall survival than the latter. The scoring method for neoIM was selected under the hypothesis that tumors with pronounced immunogenic regions may often employ immune evasion strategies. Conversely, tumors that lack these regions may have either inherently lacked or selectively eliminated immunogenic mutations to evade immune detection. While it might not capture all nuances of individual mutations, this immunogenicity biomarker provides a comprehensive and robust measure of a tumor’s immunogenic potential in a single variable that can be easily compared across different patients. This is in line with other recent studies that have shown a stronger correlation between response to immune checkpoint inhibition with the number of immunogenic variants rather than with mutational burden [6].

In contrast to the results of Snyder et al., neoIM was thus able to identify patients that would normally not be treated due to low TMB, but where treatment would actually kprovide clinical benefits. Given the clinical successes obtained with CPI in a broad array of cancer indications, including additional patients is arguably more important than ruling out patients, which can thus be achieved by tumor immunogenicity assessment with neoIM. Considering these results, neoIM can be a powerful tool both for improving target selection for personalized immunotherapies, especially in patients with low TMB, as well as for offering tumor immunogenicity as an improved biomarker for patient survival and response to therapy. However, further studies including more patients with low TMB and other cancer types are necessary to determine whether variant immunogenicity can considered a universal biomarker in low TMB tumors.

## 5. Conclusions

In summary, neoIM represents a critical step forward in computational neoantigen prediction, offering a more precise, first-in-class approach to identifying clinically relevant immunogenic peptides. By addressing the high false-positive rate plaguing current selection strategies, it has the potential to significantly accelerate the development of personalized cancer immunotherapies and improve patient outcomes.

## Figures and Tables

**Table 1:** Sources of peptides included in the benchmarking dataset.

Year of Publication	PMID	Source	Non-Immunogenic Peptides	Immunogenic Peptides
2011	21918184		477	42
2018	29397015		100	26

**Table 2:** Comparison of immunogenicity prediction tools.

	HLA Dependency	Positive Data	Negative Data	Predictive Parameters	ROC AUC—Viral (Neoantigen) Dataset	AP—Viral (Neoantigen) Dataset
neoIM	Input peptides should be presented.	Positive T-cell assay	Non-self MS-eluted ligands	Amino acid physicochemical properties.	0.80 (0.81)	0.58 (0.61)
IEDB_imm	Input peptides should be presented.	Positive T-cell assay	Negative T-cell assay	Enrichment of an amino acid in immunogenic peptides.	0.60 (0.53)	0.20 (0.39)
antigen.garnish Click or tap here to enter text.	Input peptides should be presented.	Positive T-cell assay	Self-proteome	Similarity (BLAST) to IEDB epitopes or non-mutated proteome.	0.61 (0.58)	0.20 (0.32)
PRIME Click or tap here to enter text.	Final score dependent on single HLA subtype.	Positive T-cell assay	Negative T-cell assay + random peptides	MHC affinity, amino acid frequencies at TCR-contact positions.	0.64 (0.61)	0.20 (0.35)
INeo-Epp Click or tap here to enter text.	Final score dependent on single HLA subtype.	Positive T-cell assay	Negative T-cell assay	Amino acid physicochemical property, EL rank (%), peptide entropy.	NA (0.67)	NA (0.43)

## Data Availability

The data used to support the findings of this study are included within the supplementary information file(s). The code is not available for commercial use but can be made available for research use upon reasonable request.

## Supplementary Materials

The following supporting information can be downloaded at: https://www.mdpi.com/article/10.3390/vaccines13080865/s1, Table S1: Summary of ELISpot validation results (XLSX). Figure S1. SHAP values of the top 10 most discriminating features of the neoIM model. SHAP values indicate the degree with which a given feature will impact the decision of a model: highly differentiated SHAP values for a given feature between two classes in a dataset indicate that this feature is an important criteria to rely on to discriminate the two classes. Pos: position, the amino-acid position at which a given feature is evaluated. Positions are one-indexed (zero-less), right-adjusted. If position is not specified the feature is peptide-wide. PC: Principal Component; of highest relevance, PC1 and PC3 relate to hydrophobicity.
