# Supplementary material for: Accelerating Neoantigen Discovery: A High-Throughput Approach to Immunogenic Target Identification
Source: Vaccines (Basel). 2025 Aug 15;13(8):865. doi: 10.3390/vaccines13080865 (PMC12390321; doi:10.3390/vaccines13080865)
Supplement: Supplementary file 1 [file vaccines-13-00865-s001.zip › vaccines-3800904-Figure S1.pdf]

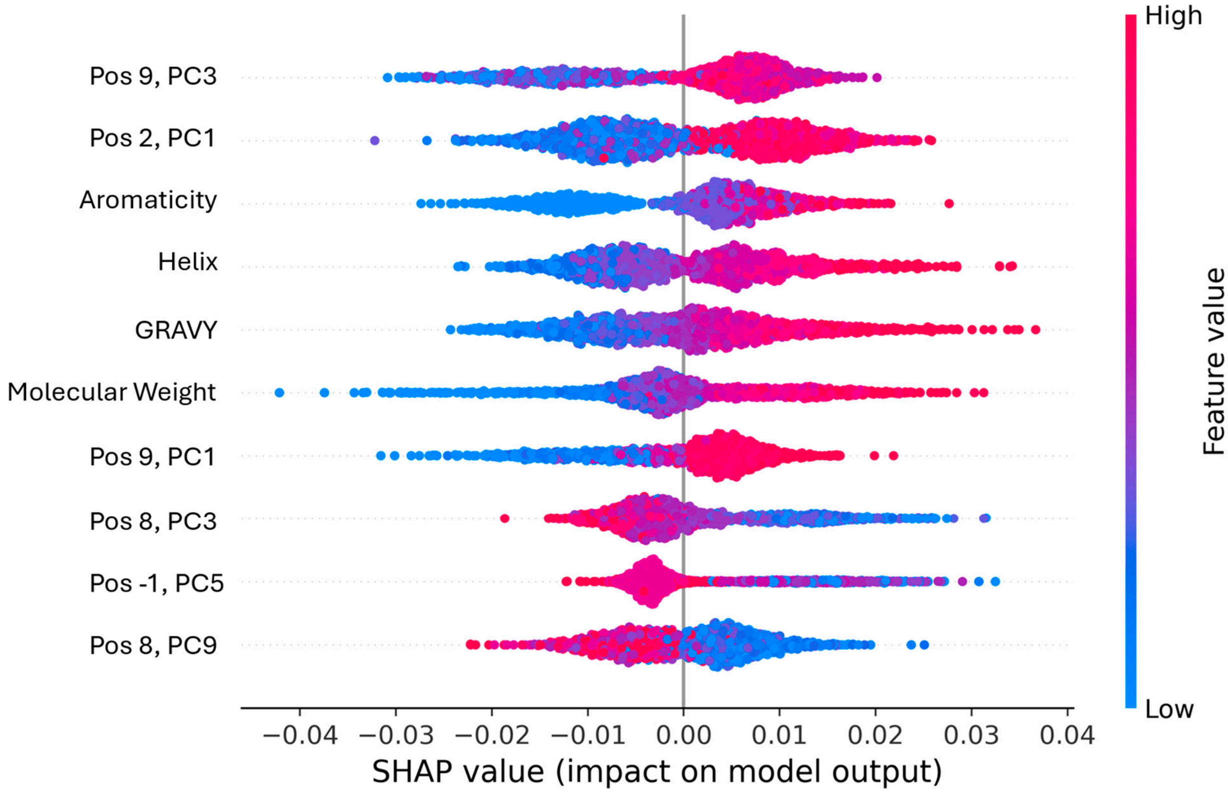

Figure S1. SHAP values of the top 10 most discriminating features of the neoIM model. SHAP values indicate the degree with which a given feature will impact the decision of a model: highly differentiated SHAP values for a given feature between two classes in a dataset indicate that this feature is an important criteria to rely on to discriminate the two classes. Pos: position, the amino-acid position at which a given feature is evaluated. Positions are one-indexed (zero-less), right-adjusted. If position is not specified the feature is peptide-wide. PC: Principal Component; of highest relevance, PC1 and PC3 relate to hydrophobicity.
